# The dilemma of chronic kidney disease and end-stage kidney disease following pre-eclampsia: a literature review and meta-analysis

**DOI:** 10.1007/s11255-025-04591-2

**Published:** 2025-06-07

**Authors:** Gaia Bianchi, Bruno Vogt, Matteo Bargagli, Claudia Ferrier

**Affiliations:** 1 Faculty of Medicine, University of Berne, Berne, Switzerland; 2 University Clinic of Nephrology and Hypertension, Inselspital Berne, Berne, Switzerland; 3 Nefrocentro Ticino, Lugano, Switzerland

**Keywords:** Pre-eclampsia, Healthy women, Kidney disease, Systematic review

## Abstract

**Purpose:**

Pre-eclampsia (PE) is a pregnancy-related multisystem syndrome, characterized by the sudden onset of hypertension after 20 weeks’ gestation. Although previous studies and meta-analyses found an association between pre-eclampsia and chronic kidney disease or end-stage kidney disease later in life, it remains unclear whether this relationship is causal. Furthermore, research conducted to date has not consistently excluded women with chronic hypertension and/or kidney disease prior to pregnancy, indicating a possible selection bias. Therefore, we undertook a systematic review of the updated literature on renal outcomes in women who were healthy prior to pregnancy and experienced pre-eclampsia.

**Methods:**

We searched PubMed-MEDLINE and Embase for eligible studies. We included retrospective and prospective studies involving healthy women with pre-eclampsia and reporting kidney outcomes. Of the 2,796 titles originally screened, 9 studies met our inclusion criteria. A random effects meta-analytic model was used for statistical analysis.

**Results:**

A statistically significant increase in the risk of developing chronic kidney disease and end-stage kidney disease later in life following pre-eclampsia was found (meta-analytic risk ratios [95% confidence interval]: 1.83 [1.16–2.89] and 8.96 [4.94–16.23], respectively), with high statistical heterogeneity. However, the only prospective study did not find a significant association between pre-eclampsia and chronic kidney disease.

**Conclusions:**

Although a significant association was identified, its clinical relevance and causality remain unclear. Postpartum medical investigation in women affected by pre-eclampsia is essential, but long-term follow-up may not be indicated in the absence of underlying conditions. Only prospective studies could clarify the relationship between pre-eclampsia and kidney disease in women who were healthy before pregnancy.

**Supplementary Information:**

The online version contains supplementary material available at 10.1007/s11255-025-04591-2.

## Introduction

Pre-eclampsia (PE) is a pregnancy-related multisystem syndrome, characterized by sudden onset of hypertension after 20 weeks of gestation, accompanied by at least one complication, such as proteinuria and/or maternal organ or uteroplacental dysfunction. The definition of PE has evolved over time, and currently, the presence of proteinuria is no longer considered to be essential for its diagnosis. Although the etiology of PE remains unclear, it is well-established that placental ischemia plays a central role [1, 2]. Abnormal cytotrophoblast invasion of the spiral arteries during early pregnancy leads to poor placental perfusion and oxidative stress, resulting in systemic endothelial dysfunction and the release of potent vasoconstrictors and proinflammatory cytokines, with clinical manifestations including hypertension, fetal growth restriction, and kidney involvement. In kidneys, this leads to glomerular endotheliosis and possibly to permanent kidney damage [3, 4]. Although the general belief is that PE is a transient reversible syndrome, that resolves within 1 to 3 months after delivery [5], it is not entirely clear whether PE or hypertensive disorders of pregnancy (HDP) are long-term risk factors for chronic kidney diseases (CKD) and end-stage kidney diseases (ESKD). Several studies and meta-analyses have shown an association between PE or hypertensive disorders of pregnancy (HDP) and subsequent development of cardiovascular, metabolic, and renal diseases [1, 3, 6–8]. However, whether this association is causal remains uncertain, partly because most available studies did not exclude women with pre-existing chronic hypertension and/or CKD, potentially introducing a selection bias due to the inclusion of independent risk factors before the index pregnancy. To address this, we focused on studies that either excluded women with such pre-existing conditions or conducted subgroup analyses on previously healthy women. Moreover, a more recent prospective study not included in previous analyses found no association between PE and CKD [9], justifying the need for an updated review.

To address the knowledge gaps mentioned above, we conducted an updated systematic review and meta-analysis of both retrospective and prospective studies, focusing on the association between PE and CKD/ESKD in women known to be healthy prior to pregnancy.

## Methods

We followed the MOOSE consensus statement on the conduct of meta-analysis of observational studies [10].

### Data sources and eligibility (PICOS) [11] criteria

**Fig. 1:**
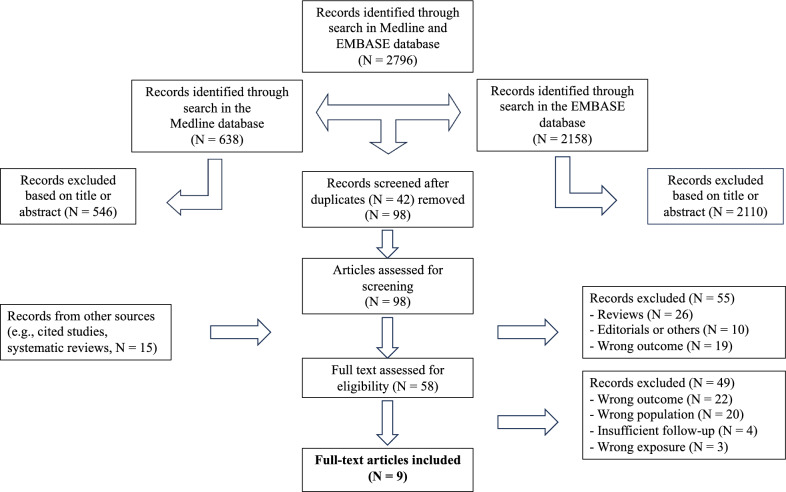
Study selection process

We conducted a review of the literature in PubMed-MEDLINE and Embase databases (2000–2024) for studies in English, French, German, and Italian. The retrieval strategy is detailed in Supplementary Material 1. Articles identified through the search were screened by title and abstract, and potentially eligible studies were further assessed according to our eligibility criteria. No search for unpublished data was conducted. The study selection process is outlined in Fig. 1 and was performed by the main author.

The eligibility criteria and PICOS criteria are as follows:

- P (patients): healthy women who had ≥ 1 episodes of PE during ≥ 1 pregnancy
- I (interventions – exposures): diagnosis of PE
- C (controls): healthy women who had at least 1 uncomplicated pregnancy in the same setting and period
- O (outcomes): long term (at least 5 years mean follow-up time) development of CKD or ESKD
- S (studies): observational and interventional studies (prospective and retrospective) with a control population available and a mean follow-up of at least 5 years, published between 2000 and 2023.

We considered women healthy if they had no known history of chronic hypertension and/or chronic kidney disease prior to pregnancy.

### Definition of exposure and outcome

The exposure (PE) was identified using reported clinical criteria, hospital record or patients self-reporting a history of PE. The outcomes (CKD or ESKD) were determined using established clinical criteria or hospital records.

The definitions of exposure and outcome were accepted as reported in the individual studies.

### Data extraction, quality, and bias assessment

The main author extracted data on number of cases and controls, study design, setting, pregnancy and study period, follow-up duration, exclusion criteria, and adjusted confounders. All the required information was directly available, so no author contact was necessary.

The quality of the evidence was assessed by the main author. Given the observational nature of the included studies, we used the National Institutes of Health (NIH) scale to evaluate study quality (Supplementary Material 2). To assess potential biases, we applied the bias classification tool developed by McDonald et al*.* [12], which evaluates six common types of bias in observational studies: selection, exposure, outcome, attrition, analytic, and confounding bias (Supplementary Material 3). Due to the retrospective nature of the analyses, no study was entirely free from bias. Nevertheless, according to the NIH scale, the studies were rated as being of medium to good quality, and each type of bias was classified as minimal to low. Clinical heterogeneity was high, mainly due to differences in study populations and/or the definitions of exposure and outcomes.

### Statistical analysis

In this meta-analysis, we assessed separately the risk of CKD and ESKD among pregnant women with and without PE. We utilized Stata software (version 16.1 and 18) to perform the analysis. Initially, risk ratios (RRs) were computed for each study to compare the incidence of CKD and ESKD in women with PE against those without. Given the substantial heterogeneity observed across studies (*I*^*2*^ = 94%, *p* < 0.001 for CKD and *I*^*2*^ = 91%, *p* < 0.001 for ESKD), a random effects model was deemed appropriate to integrate the study results. We employed the Restricted Maximum Likelihood (REML) approach for this purpose, using the **metan** function in Stata with the **random(reml)** option. This method was selected to provide a more accurate estimation by accounting for variations between the study outcomes.

### Assessment of statistical heterogeneity

Statistical heterogeneity, measured by *I*^*2*^, ranges from 0 to 100% and quantifies the degree of inconsistency across studies in a meta-analysis. A value of *I*^*2*^ < 25% is considered low, while > 75% indicates high heterogeneity [13]. In our study, despite using narrow selection criteria, we observed high statistical heterogeneity. The results must therefore be interpreted accordingly.

## Results

### Characteristics of the included studies

The initial search yielded 2,796 records. After screening titles and abstracts, 58 articles were assessed, and 9 studies met inclusion criteria. One study [14] was excluded from the statistical analysis due to the lack of raw data on healthy women, leaving 8 studies for the different meta-analyses.

The selected articles represent 8 unique study populations, providing information on 210,742 exposed and 5,951,895 controls. They were heterogeneous in terms of the number of participants, study settings, period of pregnancy, and duration of follow-up (Table 1).

**Table 1:** Characteristics of studies which investigate PE and subsequent maternal kidney disease

Source	Country, Follow-up, y	Study design, data source	Sample size, No	Exposure No	Outcome of interest, Measure of effect	Exclusion criteria	Confounders adjusted
Barrett PM [17]	Sweden ca. 21y	RC, multiple linked national registers	1,924,409	90,917	CKD, HR	Pre-pregnancy CKD, ESKD, CVD, chronic HTN, DM, SLE, Systemic sclerosis, coagulopathies, hemoglobinopathies vasculitis, multiple pregnancies, stillbirth	Maternal age, antenatal maternal BMI, education level, parity, country of origin, smoking, GDM, gestational Hypertension
Behboudi-Gandevani S [9]	Iran ca. 8y	PC, Tehran-Lipid and Glucose-Study-participants	1,851	177	CKD, HR and OR	Age 20–50, CKD, HTN, CKD + HTN, without at least 1 follow-up	Age, BMI, SBP and DBP
Kristensen JH [18]	Denmark ca. 19 y	RC, multiple linked national registers	1,072,330	1,062	CKD, HR	Age < 15, previous kidney disease, congenital/hereditary conditions with kidney defects/insufficiency, CVD, DM, HTN, AID, other conditions connected to HTN/kidney disease	Maternal age, maternal birth year, parity, history of GH and history of shortest gestation
Ayansina D [16]	Scotland ca. 30-40y	RC, multiple linked National registers	14,851	811	CKD, OR	Pre-existing HTN, kidney disease, multiples pregnancies, temporary resident	Maternal age, BMI, socio-economic status, smoking
Srialluri N [21]	USA ca. 21y	RC, Geisinger electronic health record (registry data)	27,800	2,977	CKD (eGFR < 60 ml/min), HR	CVD, DM, albuminuria, eGFR < 60 ml/min/1.73 m2 and HTN before 20 weeks of gestation	Age and year at delivery, race, number of prior deliveries, prenatal BMI, SBP and DBP, eGFR, smoking status at delivery
Wang IK [15]	Taiwan ca. 6y	RC, multiple linked national registers	240,048	17,998	ESKD, HR	Previous kidney disease, HTN, SLE, DM	Urban status, CVD, congestive heart failure, hyperlipidemia HTN and DM (during follow-up), abruption
Vikse BE [14]	Norway ca. 26y	RC, multiple linked national registers	570,433	20,918	ESKD, RR	Multiple pregnancies, essential HTN, rheumatic disease, kidney disease, DM	Year of delivery, maternal age, marital status, stillbirth, congenital malformation of the infant
Khashan AS [19]	Sweden ca. 16y	RC, multiple linked national registers	1,366,441	67,273	ESKD, HR	CKD, CVD, hypertension, or DM prior to 1rst pregnancy, multiple pregnancies	Maternal age, year of delivery, BMI, education, native country, smoking, parity
Wu CC [20]	Taiwan ca. 9y	RC, National Health Research database	944,474	8,609	ESKD, HR	DM, thrombotic micro-angiopathy, HUS, previous kidney disease, SLE, HTN secondary to renal disease	Age, delivery type, number of deliveries, and complications from delivery
Total	6,162,637 individuals (5,951,895 controls, 210,742 exposed)						

Exposure: Pre-eclampsia (PE)

*AID* autoimmune disease, *BMI* body mass index, *CKD* chronic kidney disease, *CVD* cardiovascular disease, *DM* diabetes mellitus, *eGFR* estimated glomerular filtration rate, *ESKD* end-stage kidney disease, *GDM* gestational diabetes mellitus, *GH* gestational hypertension, *HDP* hypertensive disorders of pregnancy, *HTN* chronic Hypertension, *HR* hazard ratio, *HUS* hemolytic uremic syndrome, *OR* odds ratio, *PC* prospective cohort, *PE* preeclampsia, *SBP* systolic blood pressure, *DBP* diastolic blood pressure [DBP], *RC* retrospective cohort, *RR* risk ratio, *SLE* systemic lupus erythematosus

Only one study was a prospective cohort study [9], while all the others were retrospective record-based cohort studies, most of which involved linkage between different databases.

The definitions of exposure (PE) and of outcomes (CKD, ESKD) were not homogeneous, reflecting the changes in the nomenclature of these diseases over time (Supplementary Material 4).

Other exposures of interest analyzed in addition to PE included gestational hypertension (GH) [15–17], gestational age [18], small for gestational age (SGA) [14, 17, 19], pre-existing chronic hypertension superimposed on PE [20] and the recurrence of PE in one or more pregnancies [14, 17, 19].

Besides CKD and ESKD, other outcome of interest included the risk of acute kidney failure after PE [18], risk of future hospitalization and mortality [16], as well as proteinuria and chronic hypertension [21]. Five studies assessed the association between PE and the time on set of CKD/ESKD [16–20].

As shown in Table 1, each study identified different covariates a priori as possible confounders. In some studies, additional analyses to assess the mediating effect of independent risk factors on the observed association were conducted [15, 17–20].

### Descriptive analysis: risk of CKD

Five studies analyzed the risk of developing CKD after PE, only one of which was a prospective cohort study, while the others were retrospective, record-based cohort studies. Unlike all the other studies, the prospective study by Behdoudi-Handevani et al*.* [9] did not find a statistically significant difference between the two populations (Table 2).

**Table 2:** Outcome CKD, as reported in the articles selected

Study	Cumulative incidence	Main results as reported in the articles
Barrett, PM [17]	Exposed 1,318/90,917, Not exposed 15,783/1,833,492	aHR 1.92, (95% CI 1.83–2.03), *p* < 0.001
Behboudi-Gandevani, S [9]	Exposed 41/177, Not exposed 473/1,674	aOR 1.04 (95% CI 0.77–1.40), *p* value = 0.80
Kristensen, JH [18]	Exposed 139/40,625, Not exposed 1146/1,031,675	Stratified based on delivery term^a^
Ayansina, D [16]	Exposed 61/811, Not exposed 405/10,457	aOR 1.92 (95% CI 1.45–2.56), *p* < 0.001
Srialluri, N [21]	Exposed 35/2,276, Not exposed 13/2,276	Matched cohort HR 3.23 (95% CI 1.64–6.36), *p* < 0.001

Exposure: Pre-eclampsia (PE)

*CI* confidence interval, *CKD* chronic kidney disease, *HR* hazard ratio, *OR* odds ratio, *PE* preeclampsia; RR, risk ratio

^a^ early preterm delivery aHR 2.90 (95% CI 1.70–4.96), *p* < 0.001; late-preterm delivery aHR 2.24 (95% CI 1.33–3.78), *p* < 0.001; term delivery aHR 2.27 (95% CI 1.85–2.78), *p* < 0.001

Ayansina et al*.* [16] reported low absolute risk of CKD after PE (1.3% over 2.5 million person-years of follow-up), corresponding to a hypothetical number needed to treat (NNT) of around 150 individuals. Specific analyses on subgroups of kidney diseases were conducted by Kristensen et al*.* and Barrett et al*.* [17, 18]: while a statistically significant association for hypertensive, tubulointerstitial, and diabetic CKD was found in only one study [17], both studies identified a strong association with glomerular and proteinuric disease. A stronger association with future risk of CKD was observed in the presence of PE with pre-term delivery [17, 18], SGA and recurrent PE [17].

No study has fully assessed the impact of moderating factors on the observed association between PE and future risk of CKD. However, additional analyses excluding comorbidities in the follow-up period (e.g., hypertension, cardiovascular or autoimmune diseases, and/or diabetes) showed a reduction in the strength of the association [17, 18].

The time to diagnose of CKD after the last pregnancy was found to be shorter in the exposed group [16, 17]. Additionally the association between PE and risk of developing CKD was stronger in the first five years following PE [18].

### Descriptive analysis: risk of ESKD

Four studies analyzed the risk of developing ESKD after PE, all of them being retrospective, record linkage-based cohort studies utilizing the same definition of ESKD (Table 1).

It is important to note that two studies did not exclude hypertension at baseline [20] and/or previous chronic kidney disease [14]. However, since separate analyses investigating PE in healthy women were conducted, these studies were included in our research.

All studies found a statistically significant association between exposure and outcome (Table 3).

**Table 3:** Outcome ESKD, as reported in the articles selected

Study	Cumulative incidence	Main results as reported in the articles
Wang IK [15]	Exposed 61/17,998, Not exposed 63/222,050	aHR 14.0 (95% CI 9.43–20.7), *p* < 0.001^a^
Vikse BE [14]	Exposed 67/20,918, Not exposed 410/549,515	after the first pregnancy: aRR, 3.2 (95% CI 2.2–4.5)^b^
Khashan AS [19]	Exposed 85/67,273, Not exposed 325/1,299,168	aHR 4.96 (95% CI 3.89–6.32), *p* < 0.001
Wu CC [20]	Exposed 25/8,609, Not exposed 216/933,202	aHR 9.46 (95% CI 6.10–14.68), *p* < 0.001

Exposure: Pre-eclampsia (PE)

*CI* confidence interval, *ESKD* end-stage kidney disease, *HR* hazard ratio, *PE* Preeclampsia, *RR* risk ratio

^a^ Model 2: adjusted for urban status, coronary artery disease, congestive heart failure, hyperlipidemia, and abruption

^b^ Model 2: Women with diagnosis of essential hypertension, kidney disease, rheumatic disease or diabetes mellitus before 1 st, 2nd and 3rd pregnancy were excluded. Adjusted for year of delivery, maternal age at delivery, maternal marital status, stillbirth, and congenital malformation of the infant in all included pregnancies. After the first pregnancy: aRR, 3.2 (95% CI 2.2–4.5), After the second pregnancy: pre-eclampsia in first pregnancy only aRR 2.3 (95% CI 1.3–4.1); in second pregnancy only aRR 4.7 (95% CI 2.5—9.0); in both pregnancies aRR 2.6 (95% CI 0.6—10.6), After the third pregnancy (women with >/= 3 pregnancies): pre-eclampsia in first pregnancy only aRR 5.3 (95% CI 3.0—9.6); in >/= 2 pregnancies aRR 3.0 (95% CI 0.4—21.9)

Specific analyses of kidney disease subgroups were conducted by Khashan et al*.* [19] who found a stronger association for interstitial nephritis (aHR 10.54 (95% CI 4.09–27.13) *p* < 0.001) and diabetic nephropathy (aHR 9.60 (95% CI 5.27–17.51), *p* < 0.001) than for other renal diseases. These authors performed an additional analysis after exclusion of ADPKD, which resulted in a smaller association with future ESKD risk (HR 4.50 (95% CI 3.37–5.93) *p* < 0.001) compared to the previous results.

A stronger association for future risk of ESKD was observed in the presence of PE with pre-term delivery, SGA, and recurrent PE [14, 19], while a statistically significant association was also found between maternal age at delivery and the future risk of developing ESKD [20].

Similar to CKD, no study investigating the risk of ESKD after PE has fully assessed the role of moderating risk factors in the observed association, but excluding hypertension developed during follow-up resulted in its attenuation [15]. The time to diagnose of ESKD was found to be shorter in the exposed group [20].

### Meta-analysis: CKD and ESKD

Nine studies provided effect estimates for PE and CKD (5 studies) or ESKD (4 studies). One study [14] was excluded from the analysis due to lack of raw data on healthy women.

**Fig. 2:**
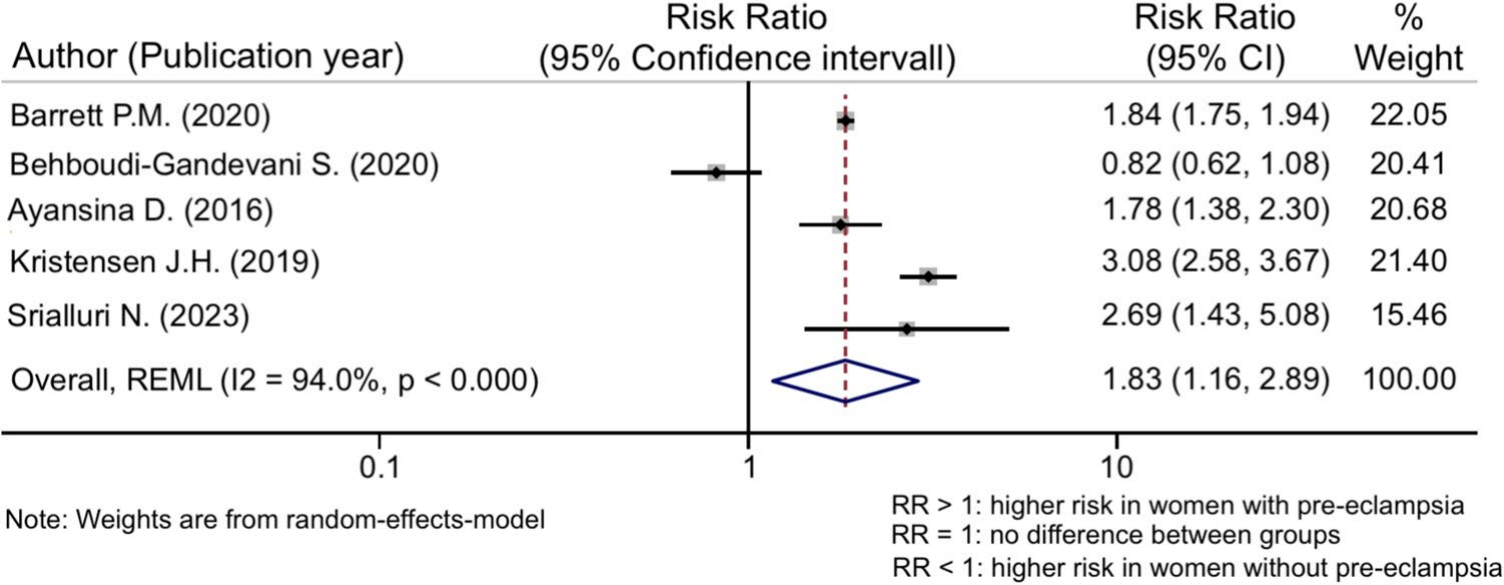
Meta-analysis of the risk for CKD in pre-eclampsia

**Fig. 3:**
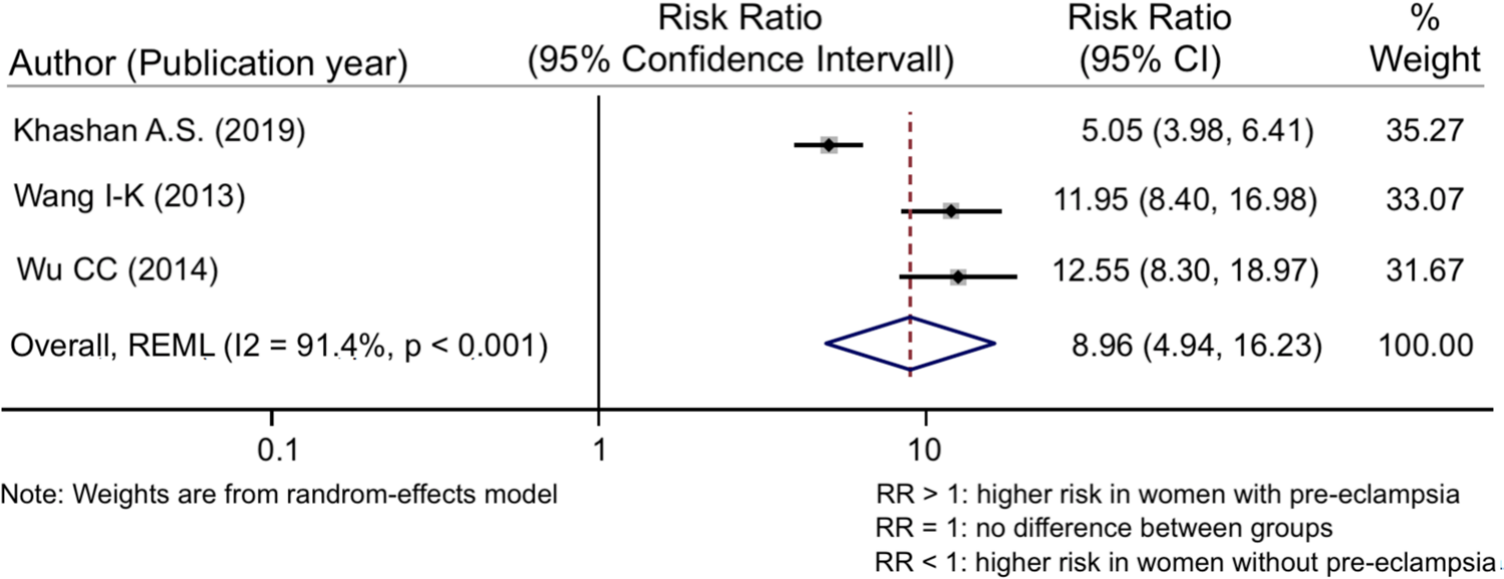
Meta-analysis of the risk for ESKD in pre-eclampsia

The risk ratio for PE and CKD was 1.83 (95% CI 1.16–2.89) (Fig. 2) while the one for PE and ESKD was higher with an overall RR of 8.96 (95% CI 4.94–16.23) (Fig. 3). Both associations were statistically significant (*p* < 0.001) but showed high levels of heterogeneity (*I*^*2*^ = 94% for CKD and *I*^*2*^ = 91% for ESKD), influencing therefore the generalization of the findings.

## Discussion

While PE has long been considered a transient and reversible condition that typically resolves within a few months postpartum, increasing evidence suggests a potential long-term impact on maternal health. The relationship between PE and cardiovascular or renal disease is complex and likely bidirectional as these conditions share a common underlying mechanism: endothelial cell damage, which is widely accepted as a key factor in the pathogenesis of PE. Consequently, pre-existing disorders characterized by endothelial dysfunction—such as hypertension, CKD, diabetes, and connective tissue or vascular diseases—may predispose women to PE [5]. On the other hand, PE, along with gestational hypertension and gestational diabetes, may itself promote vascular endothelial dysfunction, arterial stiffness, and arteriosclerosis [22], thereby contributing to long-term renal damage and increasing the risk of CKD [23, 24]. Given this complex interplay, whether women who experience HDP have an increased long-term risk of developing CKD and ESKD remains unclear.

To address this issue, we analyzed the published and updated literature on the association between PE and the future risk of maternal CKD/ESKD later in life, focusing only on women who were healthy prior to pregnancy. Since PE can be associated with reversible acute kidney injury [25, 26], we included only studies with medium- to long-term follow-up. Our analyses revealed a statistically significant increase in the risk of CKD and ESKD later in life following PE, with the risk of developing ESKD being unexpectedly higher than that of developing CKD. However, high statistical heterogeneity was observed**,** and the only prospective study did not find a significant association between PE and CKD [9].

One important finding of our review is the discrepancy between the prospective and retrospective studies. This inconsistency is likely caused by several methodological differences. Retrospective studies are more prone to misclassification and selection bias, while prospective studies often apply more rigorous exclusion criteria and control for confounding factors. Furthermore, in our specific case, the prospective study by Behboudi-Gandevani et al. had a shorter follow-up period, which may have led to an underestimation of the association with CKD.

The considerable statistical heterogeneity we observed is not unexpected and mirrors findings from previous meta-analyses in this field [1, 7, 8]. It likely reflects methodological (e.g., different exposure and outcome definitions) and clinical (e.g., different populations) variability across studies investigating long-term renal outcomes after preeclampsia. Subgroup or sensitivity analyses—such as excluding studies with broader CKD definitions or shorter follow-up—might have clarified some sources of heterogeneity. However, these would have required excluding the only available prospective study, [9], reducing methodological diversity. Moreover, given the limited number of ESKD studies, similar analyses could not be conducted without compromising statistical robustness.

The higher relative risk of ESKD compared to CKD may be partly explained by the studies’ limitations. While ESKD is uniformly defined, the definition of CKD was not consistent across the analyzed studies and varied over time. In addition, unexplored risk factors for CKD/ESKD [27–29], such as cardiovascular disease, hypertension, and diabetes, developed during follow-up may have confounded the observed association. Where performed, statistical analyses adjusting for these factors have shown a significant reduction in the strength of the association between PE and the future risk of either CKD or ESKD [15, 17, 18]. Additionally, other independent risk factors, such as obesity and smoking, as well as obstetric complications should be considered [14, 17, 19, 30, 31]. Furthermore, few studies took the etiology of renal disease into account. Women with undiagnosed congenital or hereditary kidney diseases (e.g., ADPKD) prior to pregnancy were possibly included in the analyses, leading to an overestimation of the observed association, as previously shown by Khashan et al*.* [19]*.* By adjusting and/or excluding more confounders, the strength of the observed association between PE and CKD/ESKD could be further reduced.

Preeclampsia is increasingly regarded as a stress test which may unmask an underlying maternal pathology, such as kidney disease. Proteinuria documented before pregnancy or < 20 weeks’ gestation, persistent proteinuria and/or eGFR impairment after 6 to 8 weeks postpartum all suggest pre-existing undetected renal disease [32, 33], leading to a biased attribution of causality between PE and CKD/ESKD later in life. In fact, Kristensen et al*.* [19] reported a stronger association between PE and future risk of CKD within five years of the latest pregnancy, while the strength of the association decreased with time (aHR 6.11 (95% CI 3.84–9.72) vs aHR 2.06 (95% CI 1.65–2.50), *p* < 0.001). Based on these findings and on the literature reviewed, we support the hypothesis that PE often reveals a pre-existing subclinical maternal condition, rather than representing a de novo disease triggered by pregnancy. Nonetheless, the respective contributions of pregnancy-induced endothelial injury and pre-existing dysfunction remain difficult to disentangle and require prospective investigation.

Taken together, these findings raise the question of whether the observed association justifies long-term clinical monitoring. As shown by Ayansina et al*.* [16], the absolute risk of CKD in women with PE was low (1.3% over 2.5 million person-years of follow-up), resulting in a hypothetical number needed to treat (NNT) of approximately 150 individuals to prevent/detect a single individual with CKD. Similar findings were reported by Covella et al*.* [1] (NNT 310 for ESKD and 157 for CKD). Given these high NNTs, long-term follow-up for women who have experienced PE to detect conditions with such a low incidence may not be clinically justified.

### Implications for future research and conclusion

Endothelial damage is a common pathophysiological feature of various diseases, but it remains unclear whether such damage in PE contributes directly to the long-term risk of kidney disease. Women who have experienced PE may have undiagnosed pre-existing conditions that could potentially accelerate or exacerbate the development of CKD and ESKD. Although the data collected were carefully selected from the database, we cannot completely exclude the presence of an underlying maternal disease before pregnancy. Therefore, the question of causality remains unresolved. Further meta-analyses are unlikely to provide new insights: only well-designed prospective studies with pre-pregnancy baseline assessments–and ideally a nationwide registry–can definitively clarify this relationship.

In conclusion, we suggest that women who suffered from PE should be monitored regularly for one year postpartum. However, in the absence of any signs of renal involvement or hypertension, they should be considered clinically healthy and not at elevated long-term risk for CKD and/or ESKD. Importantly, they should not face unnecessary long-term surveillance or discrimination in insurance or healthcare access.

### Strengths and limitations

Most of the studies included in our meta-analysis had large sample size, were longitudinal and had low risk of bias. However, an underlying bias due to the mostly retrospective nature of the studies must be considered, and the possibility of bias due to misclassification of exposure, outcome, or covariate (as well as missing data in the registries used) exists in all studies selected.

Another limiting factor of our research is the possible inclusion of some studies involving populations with other conditions that may lead to long-term kidney damage, such as diabetes and/or autoimmune diseases [29, 34]. It has also been suggested that the severity of pre-pregnancy renal function impairment, rather than the etiology of the renal disease itself, is a risk factor for PE and for accelerated progression of CKD [35].

These limitations show, as reported before, the need for prospective cohort studies to finally assess the question of the causality of the relationship between PE and kidney diseases.

## Supplementary Information

Below is the link to the electronic supplementary material.

Supplementary file1 (DOCX 17 KB)

Supplementary file2 (DOCX 28 KB)

Supplementary file3 (DOCX 26 KB)

Supplementary file4 (DOCX 34 KB)

## Data Availability

All data analyzed in this study were extracted from original published articles and are publicly available.
