# Supplementary material for: The dilemma of chronic kidney disease and end-stage kidney disease following pre-eclampsia: a literature review and meta-analysis
Source: Int Urol Nephrol. 2025 Jun 7;57(12):4131–40. doi: 10.1007/s11255-025-04591-2 (PMC12575586; doi:10.1007/s11255-025-04591-2)
Supplement: Supplementary file 1 — Supplementary file1 (DOCX 17 KB) [file 11255_2025_4591_MOESM1_ESM.docx]

**The dilemma of chronic kidney disease and end-stage kidney disease following pre-eclampsia: a literature review and meta-analysis**

Gaia Bianchi ^a^, Bruno Vogt ^b^, Matteo Bargagli ^b^, Claudia Ferrier ^b, c^

^a^ Faculty of Medicine, University of Berne, Switzerland

^b^ University Clinic of Nephrology and Hypertension, Inselspital Berne, Switzerland

^c^ Nefrocentro Ticino, Lugano, Switzerland

**Correspondence:** Gaia Bianchi, University of Berne, [bianchi.gaia96@gmail.com](mailto:bianchi.gaia96@gmail.com)

### Supplement material 1: Literature research

- Years: 01.01.2000-31.12. 2024
- Language: English, French, Italian, German

**PUBMED**

Concept 1: pre-eclampsia

Keywords: pre-eclampsia [tw] OR « hypertensive disorder* of pregnanc* » [tw] OR gestosis [tw] OR « toxemia of pregnanc* » [tw]

Mesh words: "Pre-Eclampsia"[Mesh]

Concept 2: healthy women

Keywords: « healthy wom* » [tw] OR « normal wom* » [tw] OR « normotensive wom* » [tw]

Mesh words: "Female"[Mesh] "Healthy Volunteers"[Mesh]

Concept 3: chronic kidney disease

Keywords: « chronic kidney diseas* » [tw] OR « end-stage kidney diseas* » [tw] OR « renal diseas* » [tw] OR « renal outcom*» [tw] OR « renal function* » [tw]

Mesh words: "Renal Insufficiency, Chronic"[Majr]

**Used for the research**: 1 AND 2 AND 3

"Renal Insufficiency, Chronic" [Majr] OR « chronic kidney diseas* » [tw] OR « end-stage kidney diseas* » [tw] OR « renal diseas* » [tw] OR « renal outcom*» [tw] OR « renal function* » [tw]

"Female"[Mesh] OR "Healthy Volunteers"[Mesh] OR « healthy wom* » [tw] OR « normal wom* » [tw] OR « normotensive wom* » [tw]

"Pre-Eclampsia"[Mesh] OR pre-eclampsia [tw] OR « hypertensive disorder* of pregnanc* » [tw] OR gestosis [tw] OR « toxemia of pregnanc* » [tw]

**MEDLINE**

Concept 1: chronic kidney disease

1 exp chronic kidney failure/

2 chronic kidney diseas*.mp.

3 end-stage kidney diseas*.mp.

4 renal diseas*.mp.

5 renal outcom*.mp.

6 renal function*.mp.

7 **1 or 2 or 3 or 4 or 5 or 6**

Concept 2: pre-eclampsia

8 preeclampsia/

9 hypertensive disorder* of pregnanc*.mp.

10 gestosis.mp.

11 toxemia of pregnanc*.mp.

12 **8 or 9 or 10 or 11**

Concept 3: healthy women

13 female/

14 normal human/

15 healthy wom*.mp.

16 normal wom*.mp.

17 normotensive wom*.mp.

**18 13 or 14 or 15 or 16 or 17**

**Used for research:** 7 AND 12 AND 18 with limit to English, French, German or Italian, years 2000-2024

19 7 and 12 and 18

20 limit 19 to ((english or french or german or italian) and yr="2000 - 2024"))
