# Supplementary material for: The dilemma of chronic kidney disease and end-stage kidney disease following pre-eclampsia: a literature review and meta-analysis
Source: Int Urol Nephrol. 2025 Jun 7;57(12):4131–40. doi: 10.1007/s11255-025-04591-2 (PMC12575586; doi:10.1007/s11255-025-04591-2)
Supplement: Supplementary file 2 — Supplementary file2 (DOCX 28 KB) [file 11255_2025_4591_MOESM2_ESM.docx]

**The dilemma of chronic kidney disease and end-stage kidney disease following pre-eclampsia: a literature review and meta-analysis**

Gaia Bianchi ^a^, Bruno Vogt ^b^, Matteo Bargagli ^b^, Claudia Ferrier ^b, c^

^a^ Faculty of Medicine, University of Berne, Switzerland

^b^ University Clinic of Nephrology and Hypertension, Inselspital Berne, Switzerland

^c^ Nefrocentro Ticino, Lugano, Switzerland

**Correspondence:** Gaia Bianchi, University of Berne, [bianchi.gaia96@gmail.com](mailto:bianchi.gaia96@gmail.com)

### Supplement material 2: National Institutes of Health scale Quality Assessment Tool for Observational Cohort and Cross-Sectional Studies

Type of answers: yes or no

CD, cannot determine; NA, not applicable; NR, not reported

| **Author, year** | **Research objective clearly stated** | **Study population clearly specified** | **Partecipation rate >50%** | **Homogenous population and uniform eligibility criteria** | **Sample size justification provided** | **Exposure of interest assessed prior to outcome** | **Sufficient timeframe follow up** | **Measurement of exposure levels** | **Exposure measurement and assessment clearly defined** | **Was the exposure(s) assessed more than once over time?** | **Outcome meseasures clearly defined** | **Were the outcome assessors blinded to the exposure status of participants?** | **≤ 20% losses at follow up** | **Statistical adjusted analysis** | **Quality rating** |
| --- | --- | --- | --- | --- | --- | --- | --- | --- | --- | --- | --- | --- | --- | --- | --- |
| Barrett 2020 [17] | yes | yes | NA | yes | no/NR | yes | yes | NA | yes | NA | yes | NA | yes | yes | good |
| Behboudi 2020 [9] | yes | yes | NA | yes | no/NR | yes | yes | NA | yes | NA | yes | NA | yes | yes | fair-good |
| Kristensen 2019 [18] | yes | yes | NA | yes | no/NR | yes | yes | NA | yes | NA | yes | NA | yes | yes | good |
| Ayansina 2016 [16] | yes | yes | NA | yes | no/NR | yes | yes | NA | yes | NA | yes | NA | NR | yes | good |
| Srialluri 2023 [21] | yes | yes | NA | yes | no/NR | yes | yes | NA | yes | NA | yes | NA | NR | yes | good |
| Wang 2013 [15] | yes | yes | NA | yes | no/NR | yes | yes | NA | yes | NA | yes | NA | NR | yes | good |
| Vikse 2008 [14] | yes | yes | NA | yes | no/NR | yes | yes | NA | yes | NA | yes | NA | NR | yes | good |
| Khashan 2019 [19] | yes | yes | NA | yes | no/NR | yes | yes | NA | yes | NA | yes | NA | NR | yes | good |
| Wu  2014 [20] | yes | yes | NA | yes | no/NR | yes | yes | NA | yes | NA | yes | NA | NR | yes | good |
