# Supplementary material for: The dilemma of chronic kidney disease and end-stage kidney disease following pre-eclampsia: a literature review and meta-analysis
Source: Int Urol Nephrol. 2025 Jun 7;57(12):4131–40. doi: 10.1007/s11255-025-04591-2 (PMC12575586; doi:10.1007/s11255-025-04591-2)
Supplement: Supplementary file 3 — Supplementary file3 (DOCX 26 KB) [file 11255_2025_4591_MOESM3_ESM.docx]

**The dilemma of chronic kidney disease and end-stage kidney disease following pre-eclampsia: a literature review and meta-analysis**

Gaia Bianchi ^a^, Bruno Vogt ^b^, Matteo Bargagli ^b^, Claudia Ferrier ^b, c^

^a^ Faculty of Medicine, University of Berne, Switzerland

^b^ University Clinic of Nephrology and Hypertension, Inselspital Berne, Switzerland

^c^ Nefrocentro Ticino, Lugano, Switzerland

**Correspondence:** Gaia Bianchi, University of Berne, [bianchi.gaia96@gmail.com](mailto:bianchi.gaia96@gmail.com)

### Supplement material 3: Biases assessment

| **Author, year** | | Selection | Exposure | Outcome | Confounding | Analytical | Attrition |
| --- | --- | --- | --- | --- | --- | --- | --- |
| Barrett 2020 [17] |  | minimal | low | low | minimal | minimal | NR |
| Behboudi, 2020 [9] | | minimal | minimal to low | minimal | minimal | minimal | low |
| Kristensen, 2019 [18] | | minimal | low | low | minimal | minimal | NR |
| Ayansina, 2016 [16] | | minimal | low | low | low | minimal | NR |
| Srialluri, 2023 [21] | | minimal | low | low | minimal | minimal | NR |
| Wang, 2013 [15] |  | minimal | low | low | minimal | minimal | NR |
| Vikse, 2008 [14] |  | minimal | low | low | minimal | minimal | NR |
| Khashan, 2019 [19] | | minimal | low | low | minimal | minimal | NR |
| Wu, 2014 [20] |  | minimal | low | low | low | minimal | NR |
|  |  |  |  |  |  |  |  |
| Type of answers: NR, Minimal, Low, Moderate, High | | | |  |  |  |  |
| NR = not reported | |  |  |  |  |  |  |
