# Supplementary material for: The dilemma of chronic kidney disease and end-stage kidney disease following pre-eclampsia: a literature review and meta-analysis
Source: Int Urol Nephrol. 2025 Jun 7;57(12):4131–40. doi: 10.1007/s11255-025-04591-2 (PMC12575586; doi:10.1007/s11255-025-04591-2)
Supplement: Supplementary file 4 — Supplementary file4 (DOCX 34 KB) [file 11255_2025_4591_MOESM4_ESM.docx]

**The dilemma of chronic kidney disease and end-stage kidney disease following pre-eclampsia: a literature review and meta-analysis**

Gaia Bianchi ^a^, Bruno Vogt ^b^, Matteo Bargagli ^b^, Claudia Ferrier ^b, c^

^a^ Faculty of Medicine, University of Berne, Switzerland

^b^ University Clinic of Nephrology and Hypertension, Inselspital Berne, Switzerland

^c^ Nefrocentro Ticino, Lugano, Switzerland

**Correspondence:** Gaia Bianchi, University of Berne, [bianchi.gaia96@gmail.com](mailto:bianchi.gaia96@gmail.com)

### Supplement material 4: Exposure and Outcome definition, assessment, and database

| **Study** | **PE definition or source of criteria** | **PE assessment/**  **database** | **CKD/ESKD definition** | **CKD/ESKD assessment/ database** |
| --- | --- | --- | --- | --- |
| Barrett [17] | ICD-8, 9 and 10 Criteria | Swedish Medical Birth Register and Swedish National Patient Register | ICD-8, 9 and 10 Criteria | Swedish Renal Register and National Patient Register |
| Behboudi-Gandevani [9] | validated self-reporting questionnaire^[[1]](#footnote-1)^ | validated self-reporting questionnaire | CKD defined as GFR (eGFR) < 60 mL/min/1.73m^2^ | Teheran lipid and glucose study |
| Kristensen [18] | ICD-8 and 10 Criteria | Medical Birth and National Patient Register of Denmark | ICD-8 and 10 Criteria | National Patient Register or the Causes of Death Register of Denmark |
| Ayansina [16] | The classification and definition of the hypertensive disorders of pregnancy (Davey and MacGillivray), 1988^[[2]](#footnote-2)^ | Aberdeen Maternity and Neonatal Databank | NICE, modified Kidney Disease Outcomes Quality Initiative (KDOQI) definitions^[[3]](#footnote-3)^ | Grampian Renal Biochemistry Dataset, The Scottish Morbidity Record, The Scottish Renal Registry, the National Records of Scotland Data on Deaths |
| Srialluri [21] | ICD-9 and 10 Criteria or outpatient laboratory results significant for albuminuria after 20 weeks of gestation but before delivery | Geisinger Health System | CKD defined as GFR (eGFR) < 60 mL/min/1.73m^2^ | Geisinger Health System outpatient serum creatinine and the CKD-EPI 2021 equation^[[4]](#footnote-4)^ |
| Wang [15] | ICD-9 Criteria | Taiwan’s National Health Insurance Research Database | ESKD defined as the need for long-term dialysis treatment or renal transplantation | Taiwan's catastrophic Illness Patients Database |
| Vikse [14] | ICD-8 and 10 Criteria as well as American College of Obstetricians and Gynaecologists 1990^[[5]](#footnote-5)^ | Medical Birth Registry of Norway | ESKD defined as the need for long-term dialysis treatment or renal transplantation | Norwegian Renal and national Cause of Death Registry of Norway |
| Khashan [19] | ICD-8, 9 and 10 Criteria | Swedish Medical Birth Register, Swedish National Patient Register, the Swedish Multi- Generation Register | ESKD defined as the need for long-term dialysis treatment or renal transplantation | Swedish Renal Register (SRR), Swedish Cause of Death Registry |
| Wu [20] | ICD-9 and ICD-9CM Criteria | Taiwan’s National Health Insurance Research Database | ESKD defined as the need for long-term dialysis treatment or renal transplantation | Taiwan’s National Health Insurance Research Database |

**Exposure**: pre-eclampsia. **Abbreviations:** ICD, International Statistical Classification of Diseases and Related Health Problems; CKD, chronic kidney disease; eGFR, estimated glomerular filtration rate; ESKD, end-stage kidney disease

1. Self-reported questionnaire based on following definition: onset of a BP level ≥140/90 mm Hg along with proteinuria > 0.3 g/24 h after 20 weeks’ gestation (Brown MA, Lindheimer MD, Swiet MD, Assche AV, Moutquin J-M. THE CLASSIFICATION AND DIAGNOSIS OF THE HYPERTENSIVE DISORDERS OF PREGNANCY: STATEMENT FROM THE INTERNATIONAL SOCIETY FOR THE STUDY OF HYPERTENSION IN PREGNANCY (ISSHP). Hypertens Pregnancy. 2001;20:ix–xiv. <https://doi.org/10.1081/prg-100104165>). [↑](#footnote-ref-1)
2. Davey DA, MacGillivray I. The classification and definition of the hypertensive disorders of pregnancy. *Am J Obstet Gynecol*. 1988;158:892–8. https://doi.org/10.1016/0002-9378(88)90090-7 [↑](#footnote-ref-2)
3. National Collaborating Centre for Chronic Conditions (UK). Chronic Kidney Disease: National Clinical Guideline for Early Identification and Management in Adults in Primary and Secondary Care [Internet]. London: Royal College of Physicians (UK); 2008 [cited 2025 Mar 5]. Available from: http://www.ncbi.nlm.nih.gov/books/NBK51773/ [↑](#footnote-ref-3)
4. Inker LA, Eneanya ND, Coresh J, Tighiouart H, Wang D, Sang Y, et al. New Creatinine- and Cystatin C–Based Equations to Estimate GFR without Race. *N Engl J Med.* 2021;385:1737–49. https://doi.org/10.1056/nejmoa2102953 [↑](#footnote-ref-4)
5. National High Blood Pressure Education Program Working Group Report on High Blood Pressure in Pregnancy. *Am J Obstet Gynecol.* 1990;163:1691–712. https://doi.org/10.1016/0002-9378(90)90653-o [↑](#footnote-ref-5)
